# Lung Ultrasound Signs and Their Correlation With Clinical Symptoms in COVID-19 Pregnant Women: The “PINK-CO” Observational Study

**DOI:** 10.3389/fmed.2021.768261

**Published:** 2022-01-21

**Authors:** Luigi Vetrugno, Alessia Sala, Daniele Orso, Francesco Meroi, Sebastiano Fabbro, Enrico Boero, Francesca Valent, Gianmaria Cammarota, Stefano Restaino, Giuseppe Vizzielli, Rossano Girometti, Maria Merelli, Carlo Tascini, Tiziana Bove, Lorenza Driul, The PINK-CO study investigators, Lisa Mattuzzi

**Affiliations:** ^1^Department of Medicine, University of Udine, Udine, Italy; ^2^Department of Anesthesia and Intensive Care Medicine, ASUFC University-Hospital of Friuli Centrale, Udine, Italy; ^3^Department of Gynecology and Obstetrics, ASUFC University-Hospital of Friuli Centrale, Udine, Italy; ^4^Anesthesia and Intensive Care Unit, San Giovanni Bosco Hospital, Turin, Italy; ^5^Department of Epidemiology and Public Health, ASUFC University-Hospital of Friuli Centrale, Udine, Italy; ^6^Department of Anesthesia and Intensive Care Medicine, University of Perugia, Perugia, Italy; ^7^Department of Radiology, ASUFC University-Hospital of Friuli Centrale, Udine, Italy; ^8^Department of Infectious Diseases, ASUFC University-Hospital of Friuli Centrale, Udine, Italy

**Keywords:** lung ultrasound, SARS-CoV-2, COVID-19, pregnant women, lung ultrasound score (LUS)

## Abstract

**Objective:**

To analyze the application of lung ultrasound (LUS) diagnostic approach in obstetric patients with severe acute respiratory syndrome coronavirus 2 (SARS-CoV-2) infection and compare LUS score and symptoms of the patients.

**Design:**

A single-center observational retrospective study from October 31, 2020 to March 31, 2021.

**Setting:**

Department of Ob/Gyn at the University-Hospital of Udine, Italy.

**Participants:**

Pregnant women with SARS-CoV-2 diagnosed with reverse transcription-PCR (RT-PCR) swab test were subdivided as symptomatic and asymptomatic patients with COVID-19.

**Exposure:**

Lung ultrasound evaluation both through initial evaluation upon admission and through serial evaluations.

**Main Outcome:**

Reporting LUS findings and LUS score characteristics.

**Results:**

Symptomatic patients with COVID-19 showed a higher LUS (median 3.5 vs. 0, *p* < 0.001). LUS was significantly correlated with COVID-19 biomarkers as C-reactive protein (CPR; *p* = 0.011), interleukin-6 (*p* = 0.013), and pro-adrenomedullin (*p* = 0.02), and inversely related to arterial oxygen saturation (*p* = 0.004). The most frequent ultrasound findings were focal B lines (14 vs. 2) and the light beam (9 vs. 0).

**Conclusion:**

Lung ultrasound can help to manage pregnant women with SARS-CoV-2 infection during a pandemic surge.

**Study Registration:**

ClinicalTrials.gov, NCT04823234. Registered on March 29, 2021.

## Keypoints

- **Question:** Are lung ultrasound findings different between pregnant women symptomatic for COVID-19 compared to those with asymptomatic COVID-19?
- **Findings:** Symptomatic patients with COVID-19 showed a higher LUS (median 3.5 vs. 0). The most frequent ultrasound findings were focal B lines (14 vs. 2) and the light beam (9 vs. 0).
- **Meaning:** Lung ultrasound is useful for evaluating pregnant women with SARS-CoV2 infection in order to establish pulmonary involvement of COVID-19.

## Introduction

Since the advent of severe acute respiratory syndrome coronavirus 2 (SARS-CoV-2) in December 2019, pregnant women have progressively become one of the most affected populations (1). Pregnant women seem to be three times at higher risk of SARS-CoV-2 infection and at increased risk for developing severe COVID-19 pneumonia than non-pregnant women (2). From January 22, 2020 to July 19, 2021, in the United States, 101,710 cases of SARS-CoV2 in pregnant women were notified, of which 17,380 cases required hospitalization and 114 were died. In total, 13.6% of patients required Intensive Care Unit (ICU) admission, and 9.4% needed invasive ventilation (2). Lung ultrasound (LUS) is non-invasive, non-ionized image, and repeatable beside tools, which gained popularity in these vulnerable populations as a diagnostic imaging tool (3). LUS is also a well-established diagnostic modality for the early diagnosis of COVID-19 pneumonia in adult patients who were admitted to the emergency department and used as a monitoring device in ICU (4). Volpicelli et al. recently identified an LUS sign that is supposed to be specific for COVID-19 (5). The “light beam” as a large hyperechoic band corresponds to the CT scan finding of ground-glass opacity, giving a high probability of COVID-19 pneumonia (6). We used a semi-quantitatively LUS score to evaluate the degree of damage and the evolution of the progression of patient disease day by day (7). Up to date, only a few case series or studies with a limited sample size have analyzed and correlated the LUS finding and the clinical signs and symptoms of pregnant patients with SARS-CoV-2 infection admitted to the hospital (8–10). The semi-quantitative assessment with an LUS score associated with the clinical symptoms could help to manage pregnant women. Our study hypothesizes that the typical LUS signs in pregnant women with COVID-19 can be easily recognized.

## Materials and Methods

### Study Protocol

We conducted a retrospective study and systematically collected data about LUS in pregnant women with SARS-CoV-2 infection who visited the Department of Gynecology and Obstetrics of the University Hospital of Udine, Italy, from October 31, 2020 to March 31, 2021.

The study was approved by the Friuli–Venezia Giulia Ethics Committee with the ID number: #3659, on February 16, 2021. The study was then registered at ClinicalTrials.gov with the ID number: NCT04823234, on March 29, 2021. All patients gave their written informed consent to manage their clinical data.

### Study Population

The inclusion criteria were (a) pregnant women with a positive nasopharyngeal molecular swab test for SARS-CoV-2 infection; (b) age > 18-y old, (c) will participate in the study. Exclusion criteria were patients with signs and symptoms of pneumonia with negative SARS-CoV-2 nasopharyngeal molecular swab test, known pulmonary autoimmune diseases, or refusal to participate in the study. Women for whom the first LUS was performed more than 7 d from symptoms onset or a positive swab were excluded.

Per an internal surveillance protocol, the pregnant patients positive for SARS-CoV2 were followed over time with check-ups at 48 h and after 5 d (except for clinical variations that required an earlier visit) or daily if they were hospitalized (for COVID-19 or other clinical reasons).

For our study, we divided patients into symptomatic for COVID-19 and asymptomatic (or carriers). We considered symptomatic patients if they developed any of the symptoms described in the literature as a possible association with COVID-19 during the follow-up or hospitalization period.

### LUS Examination

The LUS was performed by an intensive care physician with more than 10 y of experience in LUS (LV) and a resident in Obstetrics and Gynecology (AS) with a Logiq e ultrasound machine (GE Healthcare, Chicago, IL, USA) and convex probe (2–5 MHz). Online 1-wk training was set up to test the LUS inter-operator variability in interpreting LUS signs and patterns, with dedicated lessons and a final exam based on 25 clips, such as the whole range of significant COVID-19 LUS signs. Ultrasound evaluations were performed sequentially on the same patients, and the sonographers were blinded to each other's reports. The LUS evaluations were repeated on the same patient 48 h after the first evaluation and after 5 d. The LUS assessment was performed at the first and subsequent follow-up visits or in the days following admission to the ward if the patients were hospitalized. The time intervals are part of an internal surveillance protocol for COVID-19 positive patients.

**Figure 1:**
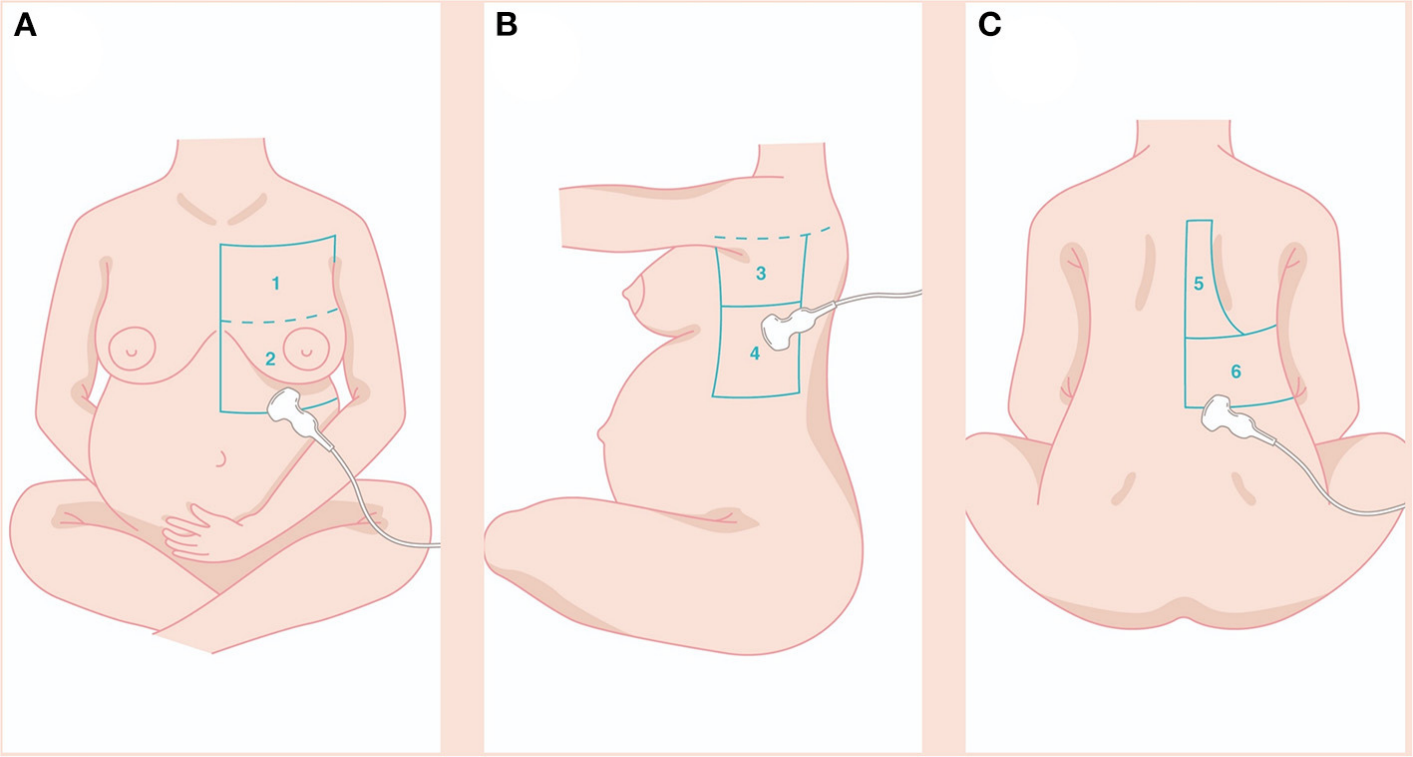
The LUS score was calculated dividing each hemithorax into six regions and represented in the specific obstetric population **(A–C)** in the following zone: anterior-superior (I), anteroinferior (II), lateral-superior (III), lateral-inferior (IV), posterior-superior (V), posterior-inferior (VI). Details in the main text. LUS, lung ultrasound.

With the patient sitting, we examined the whole pulmonary area from the upper to the basal zones anteriorly and posteriorly until the paravertebral region (Figure 1). The LUS score was calculated by dividing each hemithorax into six regions and representing in the specific obstetric population (Figures 1A–C) in the following zone: anterior-superior (I), anteroinferior (II), lateral-superior (III), lateral-inferior (IV), posterior-superior (V), and posterior-inferior (VI). The anterior axillary line and the posterior axillary line divide the thorax into the anterior, lateral, and posterior zones and a transverse line passing through the xiphoid process into the superior and inferior zones. A score from 0 to 3 was assigned to each area: 0 points for A-lines or <2 separate B lines (normal or A-pattern and presence of lung sliding); 1 point for well-spaced≥3 B lines (B-pattern and presence of lung sliding); 2 points for coalescent B-lines (light beam and presence of lung sliding); and 3 points for lung consolidation (and multiple small subpleural consolidations) (11).

The LUS score was calculated by adding all the different points to obtain a score ranging from 0 to 36. The spectrum of all LUS signs was recorded, such as (i) light beam: an artifact departing from a broad portion of the pleural line with a lucent band-form sign; (ii) separate B lines; (iii) coalescent B lines; (iv) irregular pleural line; (v) peripheral consolidations (subpleural hypoechoic images, maximum dimension 1 cm); (vi) extended consolidations (hypoechoic images with a tissue-like aspect >1.5 cm with air bronchogram, excluding compression atelectasis); and (vii) Pleural effusion.

Both operators used an N95 or higher-level mask, gown, gloves, and eye protection before entering the dedicated COVID-19 area (12).

### Data Recorded

At the first LUS examination, we recorded anthropometric parameters, such as weight, height, body mass index (BMI), gestational age, the number of fetuses, medical history, pharmacological therapy, and clinical conditions. Vital parameters, such as respiratory rate (RR), peripheral oxygen saturation (SpO_2_), PaO_2_/FiO_2_ (P/F) ratio, heart rate (HR), mean arterial pressure (MAP), body temperature (BT), and several laboratory exams were noted. The necessity of oxygen therapy, non-invasive ventilation (NIV), high-flow nasal cannula (HFNC), intubation, mechanical ventilation, and all the complications were reported with a follow-up until the discharge of the patient. We instituted a management plan to rearrange the Department of Gynecology and Obstetrics activities to meet the increasing number of admissions of pregnant women with COVID-19 (13). An intensive care physician providing regular consultation was meant to support pregnant patients at risk for admission to the intensive care unit for oxygen supplementation with NIV, HFNC, and Biphasic Positive Airway Pressure (BIPAP). In each case, the decision to move the patient to the ICU was co-managed within intensive care physician and obstetrician based on clinical criteria, such as RR > 30/min, dyspnea, SpO_2_ <90%, and P/F ratio <200 mmHg.

### Study Aim

The primary aim was to verify differences in LUS (LUS score particularly) between pregnant COVID-19 patients with symptoms and asymptomatic ones.

The secondary aims were to:

1. Verify the correlation between clinical-laboratory variables and LUS score;
2. Describe the most frequently observed ultrasound findings;
3. Verify that the clinical trend over time of patients with COVID-19 was correlated to an ultrasound evolution in terms of improvement (or worsening) of the LUS score.

### Power Analysis

Assuming that the prevalence of light beam (considered as specific LUS finding in COVID-19) among pregnant women positive for SARS-CoV-2 is similar to that observed in an international multicenter study that included 1,022 patients with positive RT-PCR tests (60%), with a case series of about 40 women hospitalized at the University Hospital of Udine, we estimate that we can reach a 95% CI with an absolute percentage accuracy of ±15% (i.e., assuming that the real prevalence is between 45 and 75%) (14).

### Statistical Analysis

We reported the data variables as the median (and interquartile range, IQR) or percentage (%) depending on if qualitative or quantitative fashion. We compared the two groups using the Kruskal-Wallis test for non-normally distributed continuous variables and the chi-squared or exact Fisher test for categorical variables. We verified the normal distribution through the Shapiro-Wilks test. We considered an alpha error ≤ of 0.05 (*p*) as statistically significant. We corrected for pairwise comparisons using the Benjamini and Hochberg method. We assessed inter-reader agreement between the two-operator using Cohen's Kappa. According to the k-value obtained, agreement was defined as slight (0–0.20); fair (0.21–0.40); moderate (0.41–0.60); substantial (0.61–0.80); or almost perfect (0.81–1).

We performed the statistical analysis using the R environment (version 4.0.3, R Foundation for Statistical Computing. Vienna, Austria) with the following packages: “compareGroups”, “readODS”, “ggplot2”, and “tidyverse”.

## Results

**Figure 2:**
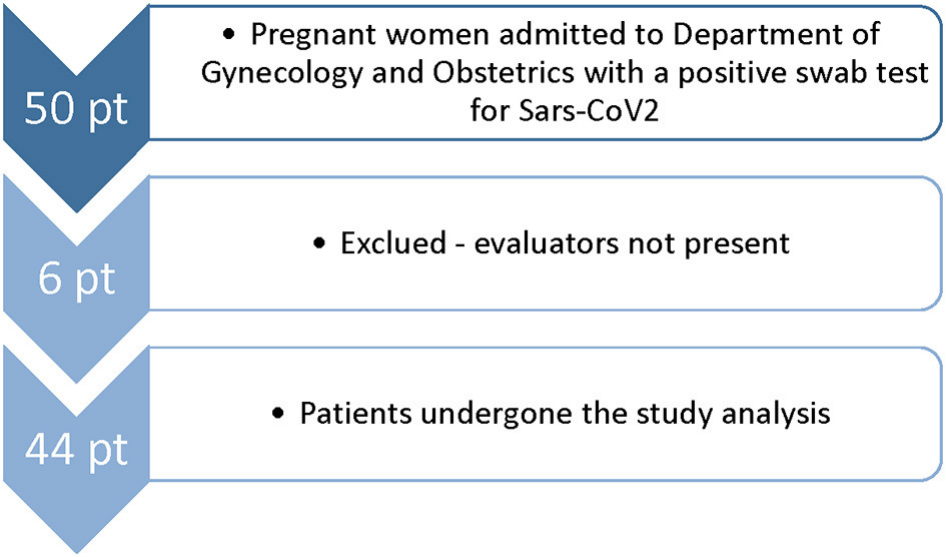
Flowchart of enrollment of COVID-19 pregnant women in University Hospital of Udine from October 31, 2020 to March 31, 2021.

During this study, 50 pregnant women who were positive for SARS-CoV-2 infection were visited at our institution, and 44 women were enrolled in the study (Figure 2). The mean age was 32 years (IQR 29–37). Twenty-four patients were symptomatic. All symptomatic patients presented at least one symptom already during the first visit. None of the patients who were asymptomatic at the first visit developed symptoms later. Median gestational age is higher in symptomatic patients (39 wk vs. 33 + 1, respectively, *p* = 0.009). The main features are described in Table 1.

**Table 1:** Distribution of the analysis variables in the sample and between the two groups, symptomatic (sCOVID-19) and asymptomatic (nsCOVID-19) patients with COVID-19.

	**Population**	**sCOVID-19**	**nsCOVID-19**	***P*-value**
	***N* = 44**	***N* = 24**	***N* = 20**	
Age (years)	32 (29–37)	35 (30–37)	31 (28–36)	0.321
Gestational age (weeks + days)	38 + 3 (31 + 5 – 39 + 3)	39 (38 + 2 – 40 + 2)	33 + 1 (25 + 3 – 38 + 5)	0.009
Twin pregnancy	4 (9.1%)	2 (8.3%)	2 (10.0%)	1.000
LOS (days)	6 (4–8)	6 (4.8–11)	6 (4–6)	0.082
Pre-pr. BMI (kg/m^2^)	23.4 (21.2–26.2)	23.4 (21.4–26.2)	23.1 (20.6–25.6)	0.612
Weight gain (Kg)	11 (7–15)	13 (8.5–15.0)	10.5 (5.8–15.8)	0.618
Smoking	7 (15.9%)	3 (12.5%)	4 (20.0%)	0.684
Comorbidity				
Obesity	3	2	1	
Gestational diabetes	7	4	3	
Arrhytmogenic dysplasia of the RV	1	1	0	
Thalassemia	1	1	0	
Hypothyroidism	5	3	2	
Cholestasis	1	1	0	
Polyhdramnios	1	1	0	
Olygodramnios	1	0	1	
Pre-eclampsia	1	0	1	
Gestational hypertension	1	1	0	
Glucose-6-phosphate dehydrogenase deficiency	1	1	0	
Thrombocytopenia	1	1	0	
Asthma	1	0	1	
Endometriosis	1	0	1	
Multiple sclerosis	1	1	0	
Symptoms				
Fever	13	13	0	
Cough	11	11	0	
Ageusia/anosmia	2	2	0	
Headache	2	2	0	
Chest pain	5	5	0	
Asthenia	4	4	0	
Dyspnea	5	5	0	
Vomit/diarrhea	2	2	0	
Bell's paralysis	1	1	0	
Pahryngodynia	1	1	0	
Muscolar pain/arthralgia	2	2	0	
CPR (mg/dL)	6.19 (3.80–12.6)	9.64 (5.17–43.1)	4.30 (2.16–6.62)	0.006
Procalcitonin (ng/mL)	0.03 (0.00–0.06)	0.03 (0.00–0.08)	0.04 (0.00–0.05)	0.948
Il-6 (pg/mL)	6.00 (3.00–13.0)	12.0 (5.00–19.0)	3.50 (2.00–5.75)	0.004
ProADM (nmol/L)	1.38 (1.09–1.75)	1.54 (1.25–1.82)	1.20 (0.99–1.53)	0.074
INR	0.92 (0.89–0.97)	0.94 (0.89–0.96)	0.91 (0.91–0.97)	0.762
APTT (sec)	1.01 (0.94–1.10)	1.08 (1.00–1.14)	0.96 (0.90–1.01)	0.004
ATIII (%)	97.0 (84.0–107)	104 (87.2–115)	87.0 (80.5–98.5)	0.025
Fibrinogen (mg/dL)	456 (405–510)	488 (432–544)	428 (373–467)	0.028
Ddimer (FEU/mL)	1,002 (763–1,488)	973 (720–1,540)	1,038 (882–1,376)	0.676
MAP (mmHg)	87.0 (77.0–93.0)	87.0 (77.0–91.5)	88.0 (77.0–93.0)	0.668
HR (bpm)	88.0 (80.0–94.0)	88.0 (79.5–96.5)	86.0 (80.2–93.8)	0.725
RR (bpm)	16.0 (15.0–17.2)	16.0 (16.0–17.2)	15.0 (14.8–17.2)	0.185
SpO_2_ (%)	98.0 (97.0–98.0)	98.0 (97.0–98.0)	98.0 (97.2–98.0)	0.296
BT (°C)	36.8 (36.5–37.4)	37.0 (36.6–37.6)	36.7 (36.5–36.8)	0.057
Oxygen th.	7 (15.9%)	7 (29.2%)	0	0.011
Mask FiO_2_ 0.4		3	0	
HFNC FiO_2_ 0.5		1	0	
LMWH				<0.001
For covid-19	12 (27.3%)	12 (50%)	0	
For other reason	5 (11.4%)	0	5 (25%)	
No LMWH	27 (61.4%)	12(50%)	15 (75%)	
Dexamethasone				0.025
For covid-19	6 (13.6%)	6 (25.0%)	0	
For other reason	1 (2.3%)	0	1 (5.0%)	
No dexamethasone	37 (84.1%)	18 (75.0%)	19 (95%)	
Antibiotic				0.058
For covid-19	5 (11.4%)	5 (20.8%)	0	
For other reason	4 (9.1%)	1 (4.2%)	3 (15.0%)	
No antibiotic	35 (79.5%)	18 (75.0%)	17 (85.0%)	
LUSS	0 (0–4)	3.5 (0–6)	0	<0.001
LU findings				
Focal B lines	16	14	2	
Light beam	9	9	0	
Pleural irregularities	5	5	0	
Consolidations	3	2	1	
Pleural effusion	1	0	1	

*LOS, length-of-stay; CPR, C reactive protein; IL6, interleukin-6; proADM, pro-Adrenomedullin; INR, international normalized ratio; aPTT, activated thromboplastin time; MAP, mean arterial pressure; HR, heart rate; RR, respiratory rate; SpO2, arterial oxygen saturation; BT, body temperature; FiO2, inspiratory fraction of oxygen; LUSS, lung ultrasound score; LMWH, low molecular weight heparin*.

The most frequent comorbidities were gestational diabetes (7/44) and hypothyroidism (5/44). We did not detect a different distribution of comorbidities between the two groups. Patients mostly reported fever (13/44) and cough (11/44). Patients with symptoms presented higher C-reactive protein (CRP; *p* = 0.006), interleukin-6 (*p* = 0.004), activated thromboplastin time (aPTT, *p* = 0.004), antithrombin (*p* = 0.025), and fibrinogen (*p* = 0.028).

Regarding the therapy administered: 7 patients required oxygen therapy (7 vs. 0; *p* = 0.011). In particular, three patients required oxygen with a Venturi mask with a FiO_2_ of 0.4 and one with an HFNC (FiO_2_ 0.5, 50 L/min). The median time to oxygen therapy was 9.5 d (IQR 4–14). None of the patients required mechanical ventilation. Low-molecular-weight heparin (12 vs. 0, *p* < 0.001) and dexamethasone (6 vs. 0, *p* = 0.025) were more frequently administered to symptomatic patients with COVID-19.

**Figure 3:**
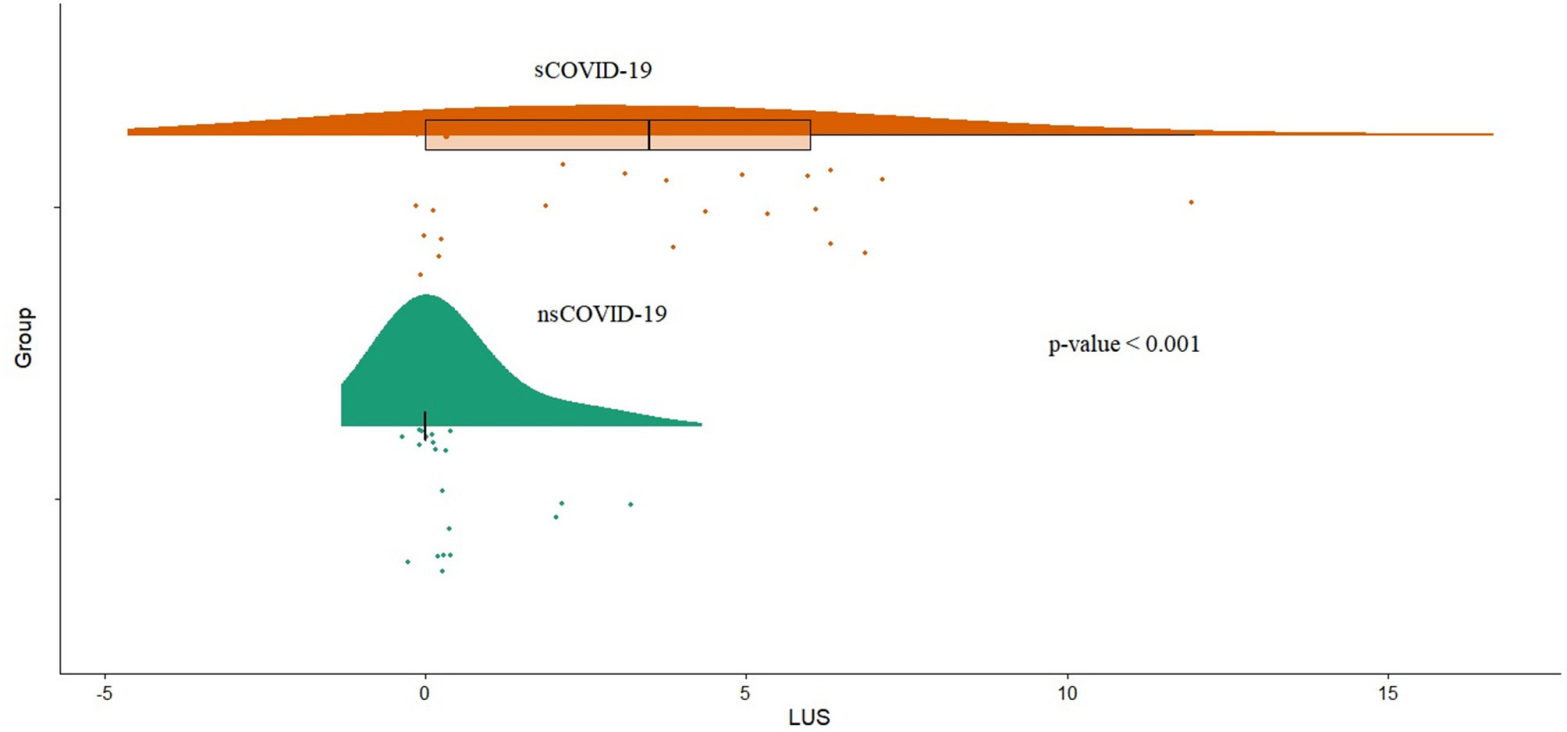
Distribution of LUS score (LUS1) values between symptomatic COVID-19 patients group (sCOVID-19) and asymptomatic group (nsCOVID-19) (*p* < 0.001). LUS, lung ultrasound.

Symptomatic patients with COVID-19 showed a higher LUS (median 3.5 vs. 0, *p* < 0.001; Figure 3). Furthermore, LUS was significantly correlated with BT (Adj*R*^2^ = 0.23; *p* = 0.004), aPTT (Adj*R*^2^ = 0.19; *p* = 0.006), D-dimer (Adj*R*^2^ = 0.22; *p* = 0.004), length of stay (Adj*R*^2^ = 0.38; *p* < 0.001), interleukin-6 (Adj*R*^2^ = 0.14; *p* = 0.013), CRP (Adj*R*^2^ = 0.14; *p* = 0.011), and pro-adrenomedullin (Adj*R*^2^ = 0.12; *p* = 0.02) and inversely related to arterial oxygen saturation (Adj*R*^2^ = 0.18; *p* = 0.004; Supplementary Figure S2).

The most frequent ultrasound findings were focal B lines (14 vs. 2) and the light beam (9 vs. 0) (Supplementary Figure S1).

**Figure 4:**
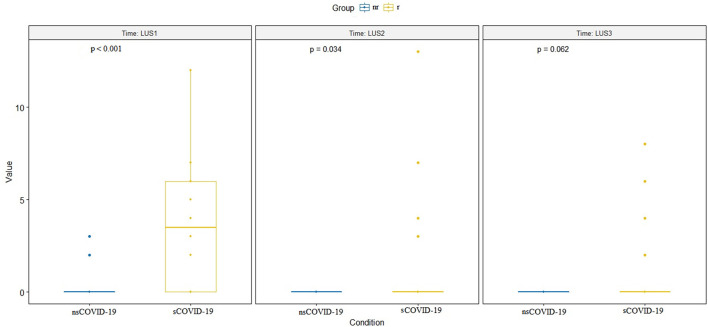
Difference between LUS values at the three times of ultrasound evaluation (LUS1 = at time zero, at admission, LUS 2 = after 2 days and LUS3 = after 7 days). The difference is significant for the first two evaluations (respectively, *p* < 0.001 and *p* = 0.034) but not for the third evaluation (*p* = 0.062). LUS, lung ultrasound.

The LUS between the two groups remained significantly different in the re-evaluations at the various subsequent times (*p* < 0.001 and *p* = 0.032, respectively), except at the last evaluation, when the symptoms had generally already disappeared (*p* = 0.062; Figure 4). Two patients underwent a further imaging step with a chest CT scan.

The 25 online questionnaires between operators showed an agreement rate of 1, the *in vivo* inter-reader agreement between the two sonographers was 0.94.

## Discussion

In the present observational study regarding pregnant women with SARS-CoV-2 infection, we used LUS as the first image modality tool to reveal lung involvement. The light beam sign was not the most common, but we found more frequently separate B-lines at the base of the lungs. In one case, the lung involvement was mono-lateral (left lung) and anterosuperior before it became bilateral. LUS score correlates well with the patient's symptomatology in patients requiring hospitalization and oxygen supplementation and with the progression of the disease anticipating the worsening or the improvement of clinical symptoms of the patients. The extension of the lung damage or the improvement could be related to the “cytokine storm” (15). Our data agree with those presented in the study of Moro et al., which first noted that the greater was the parenchymal involvement, the greater was the severity of COVID-19 pneumonia (16). Porpora et al. also found a positive correlation between LUS score and CT scan score (17). They proposed to manage patients with COVID-19 based on the clinical status and the pulmonary involvement with LUS. The literature suggests that chest radiography and CT scan as a screening tool in asymptomatic or mildly symptomatic patients without progression can be substituted by LUS. Studies have shown that in detecting parenchymal involvement in patients with COVID-19, crackles on auscultation and chest X-ray have lower sensitivity than LUS (18). In our experience, we also found it rational to monitor pregnant women with LUS score, knowing that in case of patients' worsening, chest CT scan, eventually associated with contrast enhancement, remains the goal standard diagnostic imagine modality and should not be withheld, especially before ICU admission (19). In our group, only one patient required HFNC, two patients underwent a chest CT scan, and one was admitted to the ICU. The LUS could play a crucial role in evaluating the disease progression, being CT a non-repeatable monitoring tool. Schnettler et al. also claim the same advice, studying critically ill pregnant women with COVID-19 performing LUS examinations daily (20).

There is a lack of data in the literature about interobserver agreement and reproducibility of performing LUS examination in pregnant women through different operators. Our study found a good agreement between expert and resident obstetricians after an LUS course and supervision (21). It is important to recognize that obstetricians and gynecologists represent clinicians who use ultrasound in routine practice, which is why they are technically facilitated (3). Our previous study also showed that the time needed to perform the LUS exam was low, 4.2 min (IQR 3.6–4.5) (22). Despite the personal protective equipment limiting mobility, LUS was useful and did not worsen the burden of the physicians to the clinical practice (12). Compared to what was reported by Buonsenso et al., in our series, no patient was subjected to chest X-rays, as the LUS images were already sufficiently informative and, as stated above, only two women underwent a chest CT scan image (3). The advantages of sufficient diagnostic accuracy and the absence of exposure to ionizing radiation have been sufficiently highlighted in the literature (23). During our experience, pregnant patients appear to be almost paucisymptomatic. It supposes that progesterone (a hormone with immunomodulatory properties) may have a protective role over the lungs after a viral infection (24).

The SpO_2_ was maintained not lower than 95% of the patients who underwent oxygen supplementation as recommended during pregnancy (25). Corticosteroids and anticoagulant therapy were used in some cases as infectious disease prescriptions. Dexamethasone was associated with immediate patients' sensation of symptoms improvement, and no patients were treated with Remdesivir.

Of course, some limitation in our study needs to be acknowledged. First of all, we did an observational single-center study, although we were presenting one of the largest case series in the literature in which LUS was used. Therefore, the results and the findings we have obtained need to be confirmed with a multicenter study. However, the literature reported that the learning curve of ultrasound is very rapid, even in a particular clinical setting (21–26).

Furthermore, as the study is retrospective, it was impossible to maintain complete blindness concerning the primary endpoint. We found that exploring the second quadrant anterior-inferior was difficult to explore in women with voluminous breasts. This limitation could be why LUS is highly sensitive but not specific for COVID-19 in pregnant women (27).

In conclusion, among pregnant women infected with SARS-CoV-2, our results suggest that LUS (through quantification with LUS score) correlates with the symptoms of pregnant patients with COVID-19. Diffuse B-lines and light beams were the most frequent LUS signs in symptomatic patients. LUS was a useful tool in evaluating and following up pregnant patients with COVID-19. The inter-reader agreement between the operators was optimal regardless of the experience of the various figures involved. A multicenter study urgently needs to confirm our results in an obstetric setting shortly.

## Data Availability Statement

The raw data supporting the conclusions of this article will be made available by the authors, without undue reservation.

## Ethics Statement

The studies involving human participants were reviewed and approved by Friuli-Venezia Giulia Ethics Committee with the ID number: #3659, date 16.02.2021. The patients/participants provided their written informed consent to participate in this study.

## The PINK-CO Study Investigators

Lisa Mattuzzi, MD; Matteo Marin, MD; Natascia D'Andrea, MD; Victor Zanini, MD; Michele Divella, MD; Tommaso Occhiali, MD; Elisa Barbui, midwife; Enrica Codutti, midwife; Elisa Fosca, midwife; Michela Nanino, midwife; Giuliana Scaiella, midwife, Paola Berchialla, statitician.

## Author Contributions

LV, AS, FM, and LD conceived the study and wrote the manuscript. AS and SF collected patients' data and images material. DO performed the statistical analysis and wrote the manuscript. FV performed the power analysis and supervised the manuscript. GC, MM, SR, and GV contributed to the study design and to the development of the proposal. RG, CT, TB, and LD supervised the project and approved the final manuscript. All authors approved the final version of the manuscript and agreed to submit the study protocol.

## Conflict of Interest

The authors declare that the research was conducted in the absence of any commercial or financial relationships that could be construed as a potential conflict of interest.

## Publisher's Note

All claims expressed in this article are solely those of the authors and do not necessarily represent those of their affiliated organizations, or those of the publisher, the editors and the reviewers. Any product that may be evaluated in this article, or claim that may be made by its manufacturer, is not guaranteed or endorsed by the publisher.

## Supplementary Material

Supplementary Figure S1 “Light beam” ultrasound finding at the base of a pregnant woman of 29 weeks of gestation.

Supplementary Figure S2 Linear regression between LUS score and some COVID-19 biomarkers. Counterclockwise, from top to bottom: **(A)** positive correlation between LUS score and body temperature (Adj*R*^2^ = 0.23; *p* = 0.004); **(B)** positive correlation between LUS score and aPTT (Adj*R*^2^ = 0.19; *p* = 0.006); **(C)** positive correlation between LUS score and D-dimer (Adj*R*^2^ = 0.22; *p* = 0.004); **(D)** positive correlation between LUS score and Length-of-stay (Adj*R*^2^ = 0.38; *p* < 0.001); **(E)** positive correlation between LUS score and interleukin-6 (Adj*R*^2^ = 0.14; *p* = 0.013); **(F)** positive correlation between LUS score and C reactive protein (Adj*R*^2^ = 0.14; *p* = 0.011); **(G)** positive correlation between LUS score and pro-adrenomedullina (Adj*R*^2^ = 0.12; *p* = 0.02); **(H)** inverse correlation between LUS score and arterial oxygen saturation (Adj*R*^2^ = 0.18; *p* = 0.004). LUS, lung ultrasound.

## Abbreviations

aPTT: activated partial thromboplastin time
BIPAP: Biphasic Positive Airway Pressure
BMI: body mass index
B.T.: body temperature
COVID-19: Coronavirus disease 2019
CPR: C-reactive protein
CT: computed tomography
GGO: ground-glass opacity
HFNC: high flow nasal cannula
H.R.: heart rate
ICU: Intensive Care Unit
LUS: lung ultrasound
LUSs: lung ultrasound score
MAP: mean arterial pressure
NIV: non-invasive ventilation
(P/F): PaO_2_/FiO_2_ ratio
R.R.: respiratory rate
RT-PCR: reverse transcription-polymerase chain reaction
SARS-CoV-2: severe acute respiratory syndrome coronavirus 2
SpO_2_: peripheral oxygen saturation.
