# Supplementary figures and images for: Lung Ultrasound Signs and Their Correlation With Clinical Symptoms in COVID-19 Pregnant Women: The “PINK-CO” Observational Study
Source: Front Med (Lausanne). 2022 Jan 21;8:768261. doi: 10.3389/fmed.2021.768261 (PMC8814327; doi:10.3389/fmed.2021.768261)

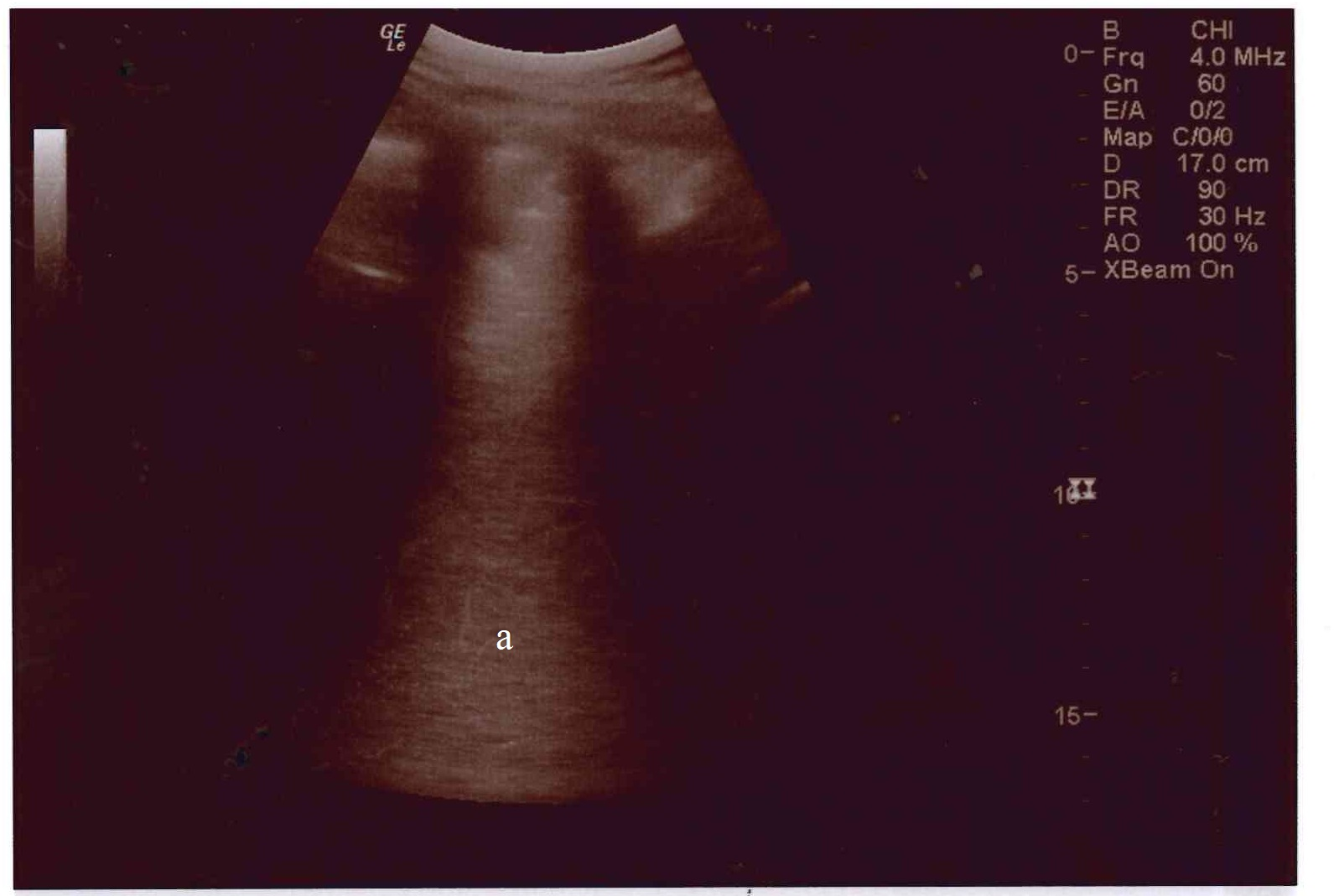

Supplement: Supplementary Figure S1 — “Light beam” ultrasound finding at the base of a pregnant woman of 29 weeks of gestation. [file Image_1.TIF]

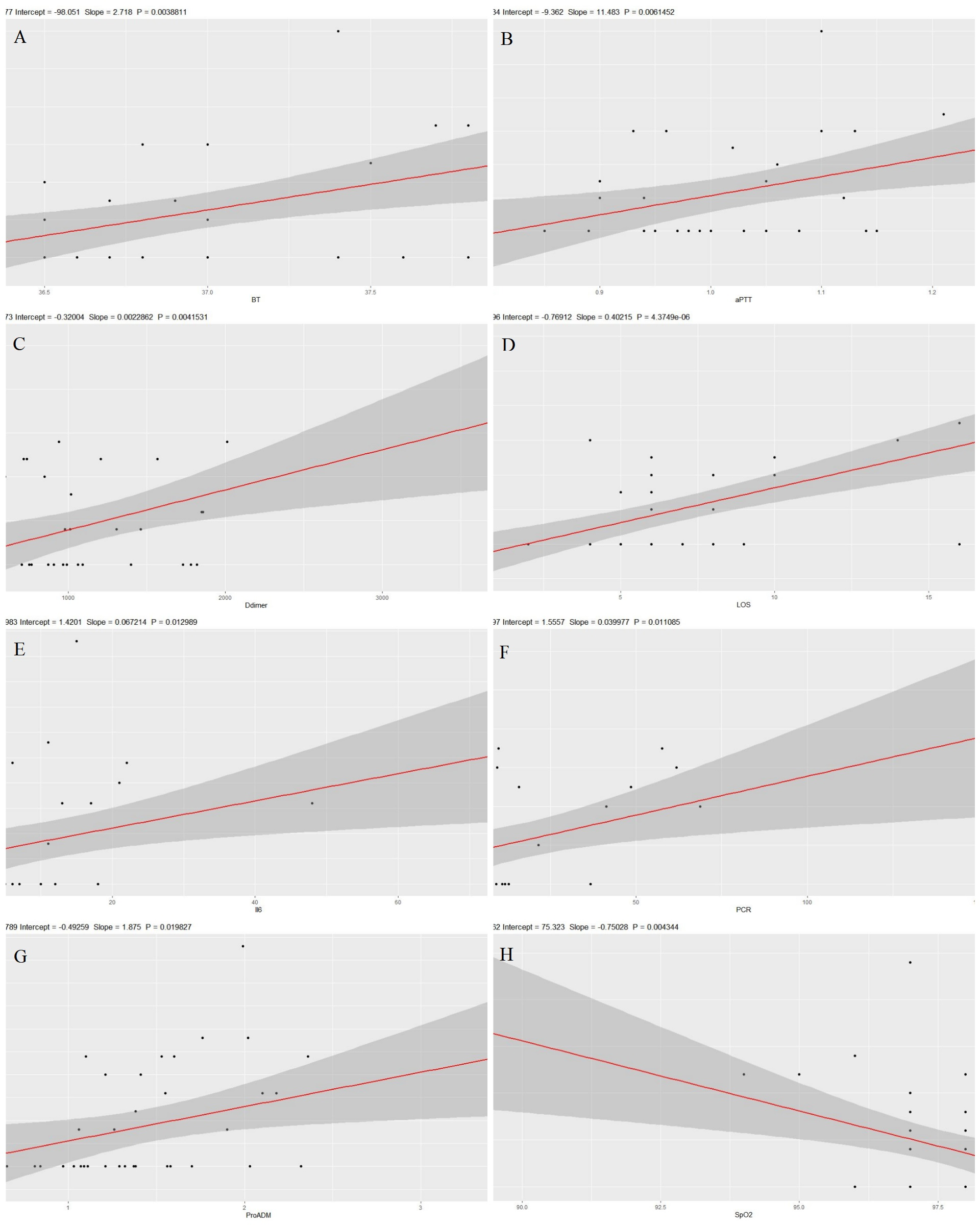

Supplement: Supplementary Figure S2 — Linear regression between LUS score and some COVID-19 biomarkers. Counterclockwise, from top to bottom: (A) positive correlation between LUS score and body temperature (AdjR2 = 0.23; p = 0.004); (B) positive correlation between LUS score and aPTT (AdjR2 = 0.19; p = 0.006); (C) positive correlation between LUS score and D-dimer (AdjR2 = 0.22; p = 0.004); (D) positive correlation between LUS score and Length-of-stay (AdjR2 = 0.38; p < 0.001); (E) positive correlation between LUS score and interleukin-6 (AdjR2 = 0.14; p = 0.013); (F) positive correlation between LUS score and C reactive protein (AdjR2 = 0.14; p = 0.011); (G) positive correlation between LUS score and pro-adrenomedullina (AdjR2 = 0.12; p = 0.02); (H) inverse correlation between LUS score and arterial oxygen saturation (AdjR2 = 0.18; p = 0.004). LUS, lung ultrasound. [file Image_2.TIF]
